# Global and Regional Estimates of Prevalent and Incident Herpes Simplex Virus Type 1 Infections in 2012

**DOI:** 10.1371/journal.pone.0140765

**Published:** 2015-10-28

**Authors:** Katharine J. Looker, Amalia S. Magaret, Margaret T. May, Katherine M. E. Turner, Peter Vickerman, Sami L. Gottlieb, Lori M. Newman

**Affiliations:** 1 School of Social and Community Medicine, University of Bristol, Bristol, United Kingdom; 2 Department of Laboratory Medicine, University of Washington, Seattle, Washington, United States of America; 3 Department of Reproductive Health and Research, World Health Organization, Geneva, Switzerland; University of Pittsburgh School of Medicine, UNITED STATES

## Abstract

**Background:**

Herpes simplex virus type 1 (HSV-1) commonly causes orolabial ulcers, while HSV-2 commonly causes genital ulcers. However, HSV-1 is an increasing cause of genital infection. Previously, the World Health Organization estimated the global burden of HSV-2 for 2003 and for 2012. The global burden of HSV-1 has not been estimated.

**Methods:**

We fitted a constant-incidence model to pooled HSV-1 prevalence data from literature searches for 6 World Health Organization regions and used 2012 population data to derive global numbers of 0-49-year-olds with prevalent and incident HSV-1 infection. To estimate genital HSV-1, we applied values for the proportion of incident infections that are genital.

**Findings:**

We estimated that 3709 million people (range: 3440–3878 million) aged 0–49 years had prevalent HSV-1 infection in 2012 (67%), with highest prevalence in Africa, South-East Asia and Western Pacific. Assuming 50% of incident infections among 15-49-year-olds are genital, an estimated 140 million (range: 67–212 million) people had prevalent genital HSV-1 infection, most of which occurred in the Americas, Europe and Western Pacific.

**Conclusions:**

The global burden of HSV-1 infection is huge. Genital HSV-1 burden can be substantial but varies widely by region. Future control efforts, including development of HSV vaccines, should consider the epidemiology of HSV-1 in addition to HSV-2, and especially the relative contribution of HSV-1 to genital infection.

## Introduction

Herpes simplex virus type 1 (HSV-1) is a highly infectious virus which is primarily transmitted by oral-oral contact and causes orolabial herpes (notably “cold sores”) in those infected[1]. The virus is highly prevalent and endemic throughout the world[2,3]. The majority of HSV-1 infections occur during childhood and infection is never cleared[1], with lifelong potential for symptomatic or asymptomatic viral shedding episodes[4,5]. In rare cases, infection can lead to more serious complications, such as encephalitis. In developed country settings, HSV-1 is the most common identified cause of sporadic encephalitis in children and adults[6,7].

HSV-2, by contrast, is almost entirely sexually transmitted, and is therefore most closely associated with genital herpes[1]. However, HSV-1 has the potential to be transmitted through oral sex to cause genital infection[1]. In a number of developed settings (e.g., the USA, Western Europe, Australia and New Zealand) there is evidence that the proportion of first episode genital herpes that is due to HSV-1 has increased, particularly among young people[8–13]. It is thought that decreases in rates of childhood infection over time[14], combined with increases in the frequency of oral sex in these populations, are driving this trend[15]. Women are more likely to acquire genital herpes than men, and this holds true for both HSV-1 and HSV-2[9,11,16,17].

The natural history of genital infection differs for the two viral types. Although first episode genital herpes is clinically indistinguishable between HSV-1 and HSV-2, subsequent recurrences are milder and much less frequent for HSV-1[5,18,19]. Neonatal herpes, a rare but devastating illness with high morbidity and mortality, can be caused by both HSV-1 and HSV-2[20]. However, a study of more than 58,000 live births showed that when mothers shed genital HSV at delivery, HSV-1 may be more likely than HSV-2 to be transmitted to the neonate[21]. In one study in Canada from 2000–2003, 63% of neonatal herpes cases were due to HSV-1[22]. This is one of a growing number of epidemiological studies that suggests that the impact of HSV-1 genital infections is widely underappreciated. It is known that HSV-2 increases HIV susceptibility and infectiousness[23–28]. The association between HSV-1 and HIV is unknown.

Determining the proportion of HSV-1 infection that is oral versus genital is difficult. Standard type-specific serological tests measure the presence of IgG antibodies to distinguish HSV-1 and HSV-2 infections, but can only tell us whether an individual is infected and not the site of infection[29]. Viral shedding (both symptomatic and asymptomatic) occurring at the site of infection can be detected by a variety of methods. However, viral shedding is episodic. This means that viral shedding studies may not detect viral presence without multiple samples being taken from each person over time[30]. Nevertheless, type-specific viral detection studies remain the most accurate way to assess the site of infection.

Quantifying the overall burden of HSV-1 infection, the burden of genital HSV-1, and the relative contribution of HSV-1 versus HSV-2 to genital herpes allows us to appropriately target prevention and treatment resources and tailor prevention counselling. In addition, such data can guide appropriate development of future interventions. The World Health Organization (WHO) has generated estimates of the global burden of HSV-2 twice: for 2003[16] and for 2012[17]. The global burden of HSV-1 infection has never been estimated to our knowledge. In this paper we present first WHO estimates of the burden of prevalent (existing) and incident (new) HSV-1 infection in 0–49 year olds for 2012 both globally and by WHO region. We also estimate the burden of genital HSV-1 infection in those aged 15–49 years.

## Methods

The method of estimation was multi-step and very similar to the method used to generate HSV-2 prevalence and incidence[16,17]. First we searched PubMed and EMBASE databases for studies reporting HSV-1 prevalence (any language) published from 2005 onwards that met our selection criteria. For search terms used see S1 Document. HSV-1 prevalence was defined as the percentage of individuals with type-specific IgG antibodies to HSV-1 cross-sectionally. Reference lists of key publications were also searched. Data from publications published before 2005 and extracted previously[3,16] were included if the selection criteria were met. The key inclusion criteria were: some detail of study location and some information on age. Studies were excluded if participants were selected on the basis of having a medical condition. The rationale for this was that prevalence in such individuals may not be generalizable to the general population. For further details of the selection criteria see S1 Document.

Only prevalence values from general populations were retained for the analyses. Data from specialised study populations such as men who have sex with men, STI clinic attendees and commercial sex workers were not used. Studies where enrolment was based on a particular minority subpopulation, for example, elderly Latino people in the Sacramento area, USA, were also not used. Specific regional criteria for use in the estimates were also applied depending on data availability by sex and study year for each region. In particular, only those prevalence values from studies from 2000 onwards were used, except for Africa and South-East Asia, where prevalence values from 1995 onwards were used due to poor data availability.

Next we pooled raw HSV-1 prevalence values for general populations by age for each of the 6 WHO regions (and by sex where data availability permitted this). The WHO regions are as follows: the Americas, Africa, Eastern Mediterranean, Europe, South-East Asia and Western Pacific (S1 Table). Pooling was done for the following age ranges: 0–4; 5–9; 10–14; 15–19; 20–24; 25–29; 30–34; 35–39; 40–44 and 45–49 years. A sample size of 20 or more was required for pooling. All prevalence values were adjusted for test sensitivity and specificity prior to pooling[31–33].

Lastly we fitted a model[34] to the pooled HSV-1 prevalence values to estimate smoothed HSV-1 prevalence and calibrate HSV-1 incidence, which were then applied to population sizes for 2012[35] to generate the regional burdens of HSV-1 infection at any site. We estimated the burden of genital HSV-1 infection by assigning values for the proportion of incident HSV-1 infections from age 15 years that are genital from existing literature. This proportion is difficult to determine, and to our knowledge, only two studies have been carried out to estimate it. In a prospective US study of HSV-2 seronegative individuals aged 17–79 years who were “high-risk” or in HSV-2 serodiscordant relationships, 19 had incident HSV-1 infections, of which 12 were symptomatic and the site of infection could be determined. Overall, 50% (6/12) of symptomatic incident HSV-1 infections were associated with genital lesions; the remaining symptomatic individuals had either orolabial lesions or pharyngitis[36]. In a more recent study of HSV seronegative women aged 18–30 years in the control arm of the HERPEVAC Trial for Women in the USA, with 127 total incident HSV-1 infections, 85% (28/33) of symptomatic incident HSV-1 infections were associated with either genital disease or both oral and genital disease[13]. We found no studies to estimate this proportion outside of the USA.

Uncertainty bounds around the estimates were computed which accounted for uncertainty in the underlying prevalence data. For a detailed description of the model and estimates calculation see S1 Document.

## Ethics Statement

It was not necessary to seek ethical approval for this study as this study is an analysis of existing published prevalence data and as such did not involve human participants directly. Correspondingly, no patient records or patient information were accessed.

## Results

### Data availability

**Fig 1:**
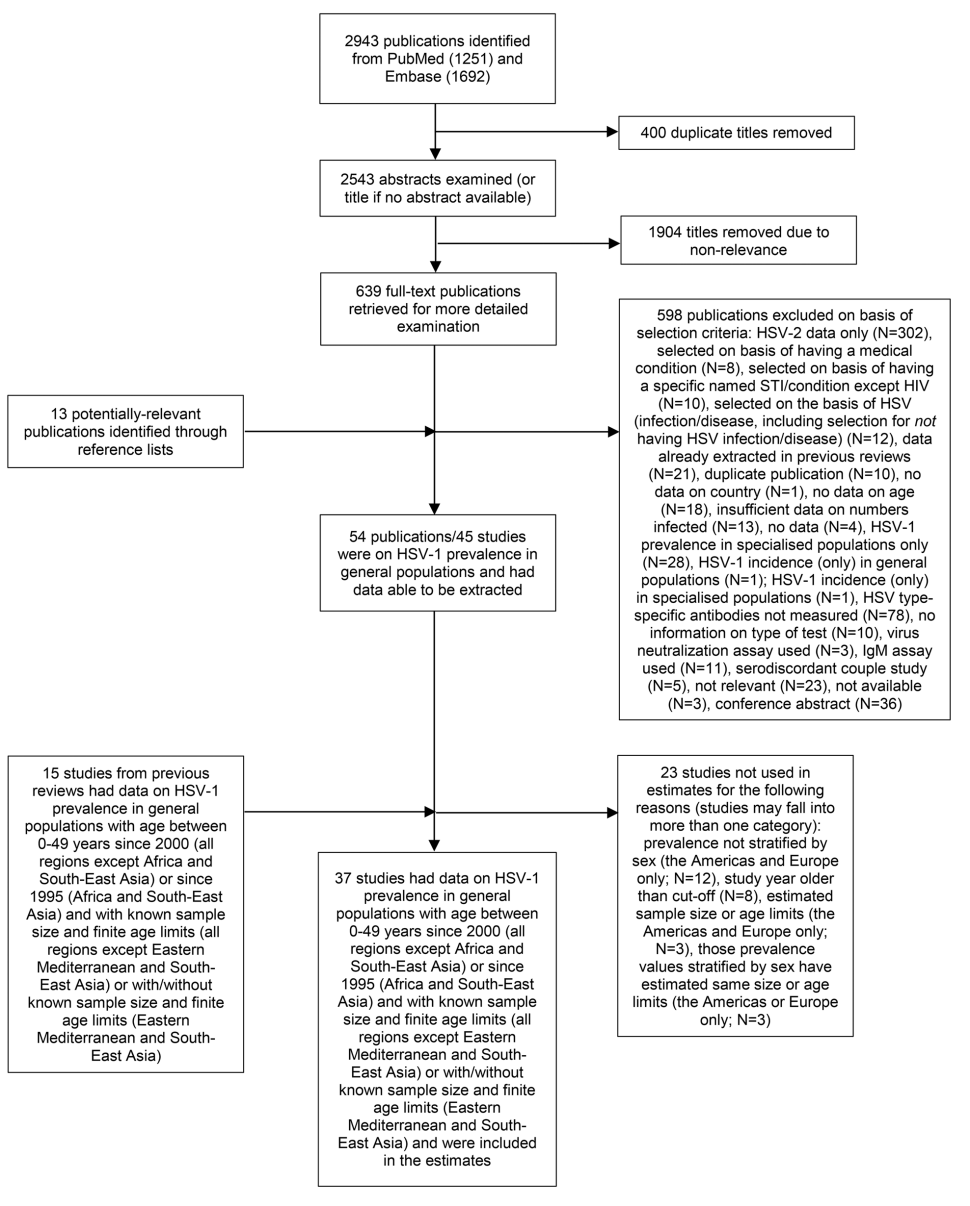
Number of studies at each stage of the literature search and study selection process.

A total of 2943 publications were identified in the literature search (Fig 1). After exclusion of 400 duplicates, the abstracts of the 2543 remaining publications were checked. This led to exclusion of a further 1904 publications due to non-relevance. The full texts of the resulting 639 publications were obtained and checked, along with 13 potentially-relevant publications identified from reference lists. Few studies reporting incidence necessitated using observed prevalence to calibrate incidence; however modelled incidences were checked against reported values and found to be in line. Fifty-four publications on 45 separate studies reported HSV-1 prevalence in general populations and met all of our general selection criteria. Of these 45 studies, 22 studies were done in populations with age between 0–49 years and met the region-specific criteria for inclusion in the estimates (S2 Table), along with a further 15 studies from earlier reviews (S1 Database and S1 Reference List).

Thirty-seven studies from 27 countries contributed 164 prevalence observations by age and a total of 40,605 individuals, of which 141 prevalence observations (34,556 individuals) were used in the estimates (S3 Table and S4 Table). Studies were represented more than once if they reported prevalence for more than one age group. Prevalence observations were more abundant for the Americas and Europe, whereas in other regions some age ranges did not have prevalence estimates. In particular, small numbers of studies in children hampered accurate model fitting for younger ages. Additionally, within-region estimates were often heterogeneous (S4 Table).

Each HSV-1 assay used was assigned a sensitivity and specificity value based on published evaluations to adjust prevalence (S5 Table). Focus Diagnostics' HerpeSelect assay (Cypress, CA) was the most commonly used assay to diagnose individuals as HSV-1 seropositive or seronegative.

### Prevalent HSV-1 infection in 2012

**Fig 2:**
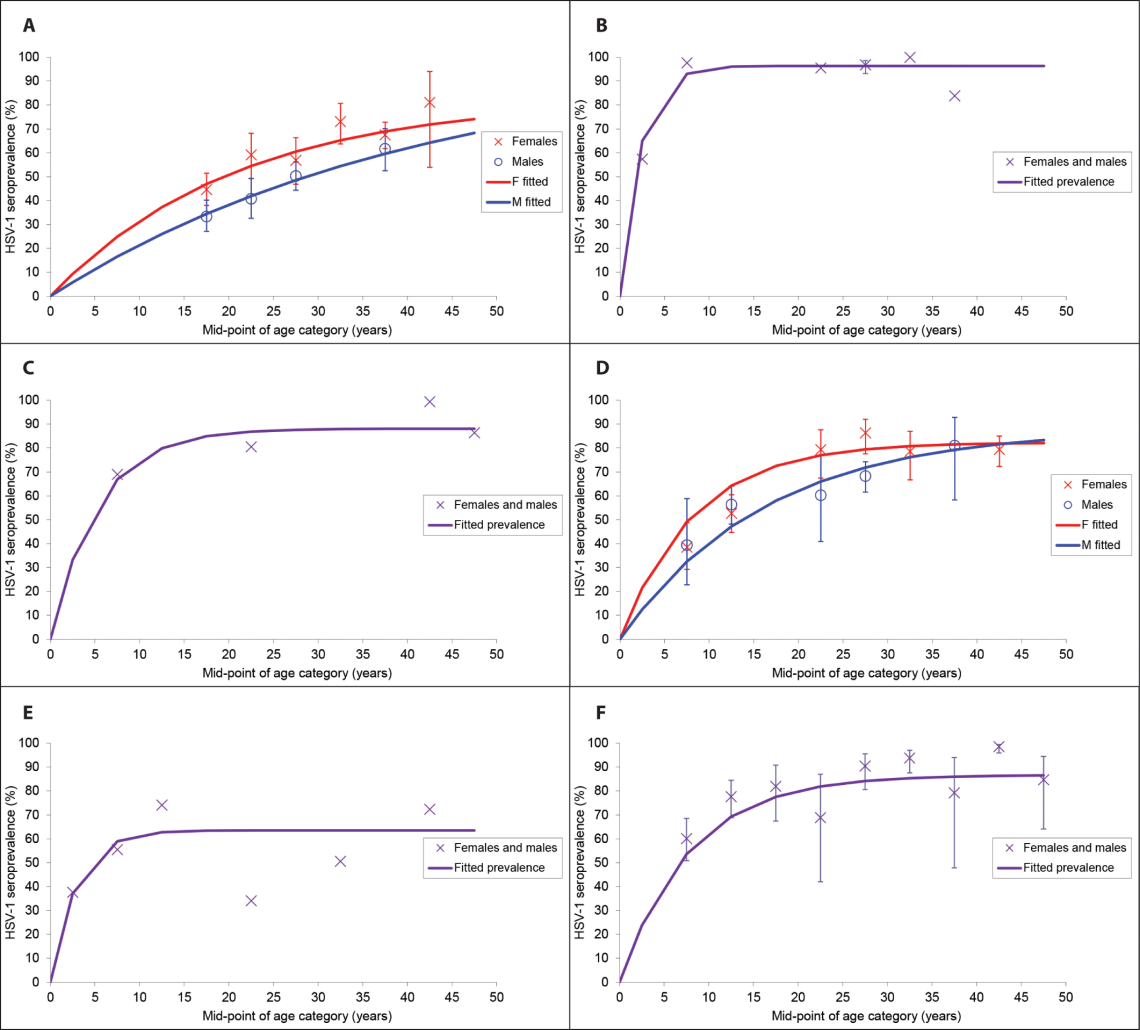
Model fits to pooled HSV-1 prevalence values by age for (A) the Americas, (B) Africa, (C) Eastern Mediterranean, (D) Europe, (E) South-East Asia and (F) Western Pacific, to generate smoothed HSV-1 prevalence and calibrate HSV-1 incidence for the HSV-1 estimates. Confidence intervals are not shown for the pooled prevalence values for those regions for which data without finite age limits and/or with estimated sample size were additionally used in the pooling (Eastern Mediterranean and South-East Asia), or where individual prevalence values were used for the model fitting in addition to pooled values (Africa).

The estimated worldwide prevalence of HSV-1 infection among 0–49 year olds in 2012 was 67% averaged across all ages (Table 1). Prevalence increased with age, and was high across all regions, but highest in Africa (87% overall prevalence) and lowest in the Americas (40–50%) (Table 1 and Fig 2). HSV-1 prevalence seemed to reach saturation by adolescence in Africa and South-East Asia, and by early adulthood in Eastern Mediterranean. In contrast, prevalence continued to increase throughout adulthood in the Americas, Europe and Western Pacific, and saturated much later, if at all (Fig 2). Overall, there were an estimated 3709 million individuals aged 0–49 years with prevalent HSV-1 infection worldwide in 2012 (Table 1). The number was highest in Africa, South-East Asia and Western Pacific, reflecting large population size.

**Table 1:** Global and regional estimates of the number of existing (prevalent) cases of HSV-1 infection in 2012 by age and sex, in millions (percentage of population with prevalent infection shown in parentheses).

**Both sexes**											
	**Age group (years)**										
	**0–4**	**5–9**	**10–14**	**15–19**	**20–24**	**25–29**	**30–34**	**35–39**	**40–44**	**45–49**	**All ages**
**Global total (all)**	**175 (27%)**	**353 (57%)**	**392 (66%)**	**420 (71%)**	**453 (74%)**	**439 (75%)**	**394 (76%)**	**378 (77%)**	**367(78%)**	**337 (79%)**	**3709 (67%)**
**Females**											
	**Age group (years)**										
**Region**	**0–4**	**5–9**	**10–14**	**15–19**	**20–24**	**25–29**	**30–34**	**35–39**	**40–44**	**45–49**	**All ages**
**Americas**	3	9	14	18	21	22	23	23	23	23	**178 (49%)**
**Africa**	36	57	52	45	40	35	29	23	18	15	**350 (87%)**
**Eastern Mediterranean**	9	20	24	25	25	24	20	16	14	12	**188 (75%)**
**Europe**	5	12	16	19	24	26	26	26	26	27	**207 (69%)**
**South-East Asia**	24	49	53	52	51	49	45	41	37	32	**432 (59%)**
**Western Pacific**	11	27	36	48	65	63	53	60	65	59	**488 (74%)**
**Global total (females)**	**86**	**175**	**194**	**208**	**225**	**219**	**196**	**188**	**183**	**168**	**1843 (68%)**
**Males**											
	**Age group (years)**										
**Region**	**0–4**	**5–9**	**10–14**	**15–19**	**20–24**	**25–29**	**30–34**	**35–39**	**40–44**	**45–49**	**All ages**
**Americas**	2	6	10	14	16	18	19	19	19	20	**142 (39%)**
**Africa**	37	58	53	46	40	35	29	23	18	15	**355 (87%)**
**Eastern Mediterranean**	9	22	25	26	27	26	22	18	15	13	**202 (75%)**
**Europe**	3	8	12	16	21	24	25	25	26	26	**187 (61%)**
**South-East Asia**	26	53	57	56	54	51	47	42	38	34	**458 (58%)**
**Western Pacific**	12	31	41	54	70	67	55	62	68	62	**521 (73%)**
**Global total (males)**	**89**	**179**	**198**	**212**	**228**	**220**	**197**	**189**	**184**	**169**	**1866 (66%)**

Totals may be slightly different due to rounding. Sex-specific estimates for Africa, Eastern Mediterranean, South-East Asia and Western Pacific were generated by applying modelled prevalence, fitted without stratification by sex, to sex-specific population sizes. Slight differences in prevalence averaged across all ages between the sexes for these regions are therefore due to different population sizes by sex.

For the Americas and Europe, for which model fitting was done separately by sex, the fitted HSV-1 prevalence was slightly lower in males compared to females (Figs 2A and 2D). This was true across all ages (except at oldest age), including childhood. The difference in children may have been in part an artefact of model fitting using limited available data: the best model fits to prevalence differences by sex among adults resulted in unequal fitted prevalence by sex among children.

### Incident HSV-1 infection in 2012

The total number of new infections in 2012 was estimated to be 118 million (Table 2). Again, the number was highest for those regions where population size was highest: Africa, South-East Asia and Western Pacific. Most HSV-1 infections occurred during the first five years of life in Africa and South-East Asia, with virtually no new infection in adulthood. Around two-thirds of HSV-1 infections occurred in those aged 0–5 years in Eastern Mediterranean, half in Western Pacific, and one-third in Europe. By contrast, around half of new HSV-1 infections in the Americas occurred in those aged 15–49 years (Table 2).

**Table 2:** Global and regional estimates of the number of new (incident) cases of HSV-1 infection in 2012 by age and sex, in millions (percentage of population with incident infection shown in parentheses).

**Both sexes**											
	**Age group (years)**										
	**0–4**	**5–9**	**10–14**	**15–19**	**20–24**	**25–29**	**30–34**	**35–39**	**40–44**	**45–49**	**All ages**
**Global total (all)**	**79 (12%)**	**19 (3%)**	**8 (1%)**	**4 (0.8%)**	**3 (0.5%)**	**2 (0.3%)**	**1 (0.2%)**	**0.9 (0.2%)**	**0.7 (0.1%)**	**0.5 (0.1%)**	**118 (2%)**
**Females**											
	**Age group (years)**										
**Region**	**0–4**	**5–9**	**10–14**	**15–19**	**20–24**	**25–29**	**30–34**	**35–39**	**40–44**	**45–49**	**All ages**
**Americas**	1.351	1.082	0.865	0.681	0.529	0.406	0.302	0.224	0.167	0.128	**6 (2%)**
**Africa**	15.626	1.412	0.129	0.012	0.001	0.000	0.000	0.000	0.000	0.000	**17 (4%)**
**Eastern Mediterranean**	4.132	1.460	0.533	0.201	0.076	0.028	0.009	0.003	0.001	0.000	**6 (3%)**
**Europe**	2.148	1.103	0.583	0.348	0.221	0.128	0.068	0.036	0.020	0.011	**5 (2%)**
**South-East Asia**	10.503	1.828	0.313	0.053	0.009	0.001	0.000	0.000	0.000	0.000	**13 (2%)**
**Western Pacific**	5.078	2.480	1.295	0.802	0.531	0.265	0.116	0.068	0.039	0.019	**11 (2%)**
**Global total (females)**	**39**	**9**	**4**	**2**	**1**	**0.8**	**0.5**	**0.3**	**0.2**	**0.2**	**57 (2%)**
**Males**											
	**Age group (years)**										
**Region**	**0–4**	**5–9**	**10–14**	**15–19**	**20–24**	**25–29**	**30–34**	**35–39**	**40–44**	**45–49**	**All ages**
**Americas**	0.900	0.811	0.730	0.644	0.555	0.472	0.390	0.318	0.264	0.226	**5 (1%)**
**Africa**	16.032	1.444	0.131	0.012	0.001	0.000	0.000	0.000	0.000	0.000	**18 (4%)**
**Eastern Mediterranean**	4.360	1.544	0.564	0.212	0.081	0.030	0.010	0.003	0.001	0.000	**7 (3%)**
**Europe**	1.367	0.947	0.675	0.542	0.459	0.353	0.250	0.178	0.128	0.095	**5 (2%)**
**South-East Asia**	11.459	1.996	0.341	0.057	0.009	0.002	0.000	0.000	0.000	0.000	**14 (2%)**
**Western Pacific**	5.779	2.811	1.452	0.891	0.577	0.281	0.121	0.070	0.041	0.019	**12 (2%)**
**Global total (males)**	**40**	**10**	**4**	**2**	**2**	**1**	**0.8**	**0.6**	**0.4**	**0.3**	**61 (2%)**

Totals may be slightly different due to rounding. Sex-specific estimates for Africa, Eastern Mediterranean, South-East Asia and Western Pacific were generated by applying modelled incidence, calibrated without stratification by sex, to sex-specific population sizes. Region-specific estimates are given to 3 d.p. due to some small numbers of infected individuals, but this level of accuracy is unlikely to be supported by the model.

### Genital HSV-1 infection in 2012

An estimated 140 million people aged 15–49 years were calculated to have prevalent genital HSV-1 infection globally in 2012, assuming 50% of incident infections in this age group were genital, and up to 239 million people if 85% of new HSV-1 infections were genital (Table 3). Patterns of genital HSV-1 infection by age, sex and region were unaffected by changing the assumption of genital incident infection. The Americas and Western Pacific contributed the most genital HSV-1 cases: the Americas by virtue of highest genital HSV-1 prevalence, and Western Pacific by virtue of large population size and medium prevalence. Higher genital HSV-1 incidence was reflective of adult versus childhood acquisition. Thus, Africa and South-East Asia seemed to have negligible genital HSV-1.

**Table 3:** Global and regional estimates of the number of existing (prevalent) cases of genital HSV-1 infection among 15–49 year olds in 2012 by age and sex, in millions (percentage of population with prevalent infection shown in parentheses), as a function of the assumed proportion of incident HSV-1 infections in this age group that are genital.

**ASSUMING 50% OF INCIDENT HSV-1 INFECTIONS AMONG 15–49 YEAR OLDS ARE GENITAL**								
**Both sexes**								
	**Age group (years)**							
	**15–19**	**20–24**	**25–29**	**30–34**	**35–39**	**40–44**	**45–49**	**All ages**
**Global total (all)**	**5 (0.8%)**	**15 (2%)**	**21 (3%)**	**22 (4%)**	**25 (5%)**	**27 (6%)**	**27 (6%)**	**140 (4%)**
**Females**								
	**Age group (years)**							
**Region**	**15–19**	**20–24**	**25–29**	**30–34**	**35–39**	**40–44**	**45–49**	**All ages**
**Americas**	0.697	2.184	3.275	3.954	4.358	4.623	4.859	**24 (10%)**
**Africa**	0.013	0.022	0.020	0.017	0.013	0.011	0.009	**0.1 (0.0%)**
**Eastern Mediterranean**	0.217	0.533	0.614	0.556	0.458	0.390	0.341	**3 (2%)**
**Europe**	0.368	1.189	1.698	1.889	1.982	2.030	2.123	**11 (5%)**
**South-East Asia**	0.059	0.118	0.123	0.115	0.104	0.094	0.083	**0.7 (0.1%)**
**Western Pacific**	0.849	2.931	3.713	3.492	4.117	4.626	4.253	**24 (5%)**
**Global total (females)**	**2**	**7**	**9**	**10**	**11**	**12**	**12**	**63 (3%)**
**Males**								
	**Age group (years)**							
**Region**	**15–19**	**20–24**	**25–29**	**30–34**	**35–39**	**40–44**	**45–49**	**All ages**
**Americas**	0.651	2.108	3.279	4.091	4.608	5.018	5.455	**25 (10%)**
**Africa**	0.014	0.022	0.020	0.017	0.014	0.011	0.009	**0.1 (0.0%)**
**Eastern Mediterranean**	0.229	0.568	0.666	0.617	0.513	0.426	0.360	**3 (2%)**
**Europe**	0.558	1.986	3.109	3.726	4.137	4.437	4.737	**23 (10%)**
**South-East Asia**	0.064	0.124	0.128	0.120	0.108	0.098	0.086	**0.7 (0.1%)**
**Western Pacific**	0.943	3.186	3.930	3.630	4.252	4.820	4.427	**25 (5%)**
**Global total (males)**	**2**	**8**	**11**	**12**	**14**	**15**	**15**	**77 (4%)**
**ASSUMING 85% OF INCIDENT HSV-1 INFECTIONS AMONG 15–49 YEAR OLDS ARE GENITAL**								
**Both sexes**								
	**Age group (years)**							
	**15–19**	**20–24**	**25–29**	**30–34**	**35–39**	**40–44**	**45–49**	**All ages**
**Global total (all)**	**8 (1%)**	**25 (4%)**	**35 (6%)**	**38 (7%)**	**42 (9%)**	**45 (10%)**	**45 (11%)**	**239 (6%)**
**Females**								
	**Age group (years)**							
**Region**	**15–19**	**20–24**	**25–29**	**30–34**	**35–39**	**40–44**	**45–49**	**All ages**
**Americas**	1.184	3.712	5.568	6.721	7.409	7.859	8.261	**41 (17%)**
**Africa**	0.023	0.038	0.034	0.029	0.023	0.018	0.015	**0.2 (0.1%)**
**Eastern Mediterranean**	0.369	0.907	1.044	0.945	0.778	0.664	0.581	**5 (3%)**
**Europe**	0.625	2.022	2.887	3.211	3.370	3.450	3.609	**19 (9%)**
**South-East Asia**	0.100	0.200	0.208	0.196	0.177	0.160	0.141	**1 (0.2%)**
**Western Pacific**	1.443	4.982	6.313	5.936	7.000	7.864	7.231	**41 (8%)**
**Global total (females)**	**4**	**12**	**16**	**17**	**19**	**20**	**20**	**107 (6%)**
**Males**								
	**Age group (years)**							
**Region**	**15–19**	**20–24**	**25–29**	**30–34**	**35–39**	**40–44**	**45–49**	**All ages**
**Americas**	1.107	3.584	5.575	6.954	7.834	8.530	9.274	**43 (18%)**
**Africa**	0.023	0.038	0.034	0.029	0.023	0.018	0.015	**0.2 (0.1%)**
**Eastern Mediterranean**	0.390	0.966	1.133	1.049	0.872	0.725	0.612	**6 (3%)**
**Europe**	0.948	3.376	5.286	6.333	7.033	7.543	8.053	**39 (17%)**
**South-East Asia**	0.108	0.212	0.218	0.204	0.183	0.166	0.147	**1 (0.2%)**
**Western Pacific**	1.604	5.416	6.680	6.171	7.229	8.194	7.526	**43 (8%)**
**Global total (males)**	**4**	**14**	**19**	**21**	**23**	**25**	**26**	**131 (7%)**

Totals may be slightly different due to rounding. Region-specific estimates are given to 3 d.p. due to some small numbers of infected individuals, but this level of accuracy is unlikely to be supported by the model.

The higher overall HSV-1 prevalence on entering adolescence and earlier saturation in prevalence in females compared to males in Europe in the model fits, and to a lesser extent in the Americas, meant higher genital HSV-1 prevalence was reached among males compared to females. Again this may have been, at least in part, an artefact of fitting the model to limited available data, generating unequal prevalence in childhood.

### Genital herpes due to HSV-1 versus HSV-2 in 2012

Collating the numbers of individuals with genital HSV-1 and HSV-2 infection, and adjusting for the expected number of individuals with co-infection (if HSV-2 infection is completely protective against subsequent genital HSV-1 infection[36,37], but prior genital HSV-1 infection does not afford any protection against HSV-2 infection[2]), gave an estimate for the total, global number of individuals aged 15–49 years with prevalent genital HSV infection in 2012 of 544 million (15% prevalence) (Table 4). This calculation assumed 50% of incident HSV-1 infections in this age group are genital; assuming 85% of HSV-1 infections are genital resulted in 633 million cases (17% prevalence). Of these, an estimated 322–360 million were women and 223–273 million were men. Region-specific analysis showed that different dynamics can result in similar overall HSV prevalence (Tables 3 and 4). For example, Africa and the Americas both had high genital herpes prevalence (>19%), but for quite different reasons: prevalence of genital HSV-1 was estimated to be >9% in the Americas versus ~0% in Africa, while Africa had higher HSV-2 prevalence.

**Table 4:** Global and regional estimates of the prevalence of, and total number of individuals with, genital herpes infection due to either HSV-1 or HSV-2 among 15–49 year olds in 2012, by sex, as a function of the assumed proportion of incident HSV-1 infections in this age group that are genital.

**ASSUMING 50% OF INCIDENT HSV-1 INFECTIONS AMONG 15–49 YEAR OLDS ARE GENITAL**					
**Females**			**Males**		
**Region**	**Prevalence of genital HSV infection (%)**	**Number with HSV-1 or HSV-2 genital infection (millions)**	**Region**	**Prevalence of genital HSV infection (%)**	**Number with HSV-1 or HSV-2 genital infection (millions)**
**Americas**	26	65	**Americas**	20	48
**Africa**	38	81	**Africa**	25	54
**Eastern Mediterranean**	14	22	**Eastern Mediterranean**	5	9
**Europe**	14	32	**Europe**	14	31
**South-East Asia**	9	42	**South-East Asia**	7	34
**Western Pacific**	16	80	**Western Pacific**	9	47
**Global total (females)**	**18**	**322**	**Global total (males)**	**12**	**223**
**Global total (all)**	**544 million (15%)**				
**ASSUMING 85% OF INCIDENT HSV-1 INFECTIONS AMONG 15–49 YEAR OLDS ARE GENITAL**					
**Females**			**Males**		
**Region**	**Prevalence of genital HSV infection (%)**	**Number with HSV-1 or HSV-2 genital infection (millions)**	**Region**	**Prevalence of genital HSV infection (%)**	**Number with HSV-1 or HSV-2 genital infection (millions)**
**Americas**	32	78	**Americas**	26	63
**Africa**	38	81	**Africa**	25	54
**Eastern Mediterranean**	15.5	24	**Eastern Mediterranean**	6.5	11
**Europe**	18	39	**Europe**	21	47
**South-East Asia**	9	42	**South-East Asia**	7	35
**Western Pacific**	19	95	**Western Pacific**	12	64
**Global total (females)**	**20**	**360**	**Global total (males)**	**15**	**273**
**Global total (all)**	**633 million (17%)**				

Totals may be slightly different due to rounding. Prevalence (and corresponding numbers infected) reduced by estimated fraction of infections that are co-infections (genital HSV-1 prevalence * HSV-2 prevalence) to avoid double counting, assuming that genital HSV-1 does not occu after HSV-2 infection, and that HSV-2 infection is independent of previous infection with genital HSV-1. Note that this table is for infection, not disease.

### Uncertainty analysis

The 95% credible range for the number of individuals aged 0–49 years with any prevalent HSV-1 infection worldwide in 2012 was calculated to be 3440–3878 million: 1709–1931 million women and 1728–1965 men (Table 5). The equivalent 95% credible range for the global number aged 15–49 years with prevalent genital HSV-1 infection in 2012 was dependent on the assumed proportion of incident HSV-1 infections at these ages that were genital, and varied from 67–212 million when this proportion was 50%, and from 113–361 million when it was 85% (Table 5). The credible ranges were very wide for all regions (except Africa, for which the computed credible range was not reliable due to insufficient pooled prevalence values to sample). Thus, plausible changes in the underlying prevalence data informing the model fits can generate very different patterns of infection, including earlier saturation in overall HSV-1 prevalence and little subsequent genital HSV-1 in the Americas (although the majority of runs gave estimates for genital HSV-1 at the upper end), and later saturation in overall HSV-1 and substantial genital HSV-1 in Eastern Mediterranean and South-East Asia (although the majority of runs gave estimates for genital HSV-1 at the lower end).

**Table 5:** 95% credible bounds for the global and regional estimates of the number of cases of any prevalent HSV-1 infection, and prevalent genital HSV-1 infection, in 2012 by sex, in millions, incorporating uncertainty in the underlying HSV-1 prevalence data, and as a function of the assumed proportion of incident HSV-1 infections in this age group that are genital.

**Both sexes**						
	**Prevalent HSV-1 infection (any site)**		**Prevalent genital HSV-1 infection (assuming 50% of incident infections are genital from age 15)**		**Prevalent genital HSV-1 infection (assuming 85% of incident infections are genital from age 15)**	
	**Lower bound**	**Upper bound**	**Lower bound**	**Upper bound**	**Lower bound**	**Upper bound**
**Global total (all)**	**3440**	**3878**	**67**	**212**	**113**	**361**
**Females**						
	**Prevalent HSV-1 infection (any site)**		**Prevalent genital HSV-1 infection (assuming 50% of incident infections are genital from age 15)**		**Prevalent genital HSV-1 infection (assuming 85% of incident infections are genital from age 15)**	
**Region**	**Lower bound**	**Upper bound**	**Lower bound**	**Upper bound**	**Lower bound**	**Upper bound**
**Americas**	148	211	0.04	29.06	0.06	49.41
**Africa**	335^a^	355^a^	0.05^a^	0.14^a^	0.09^a^	0.23^a^
**Eastern Mediterranean**	152	210	0.03	13.89	0.06	23.62
**Europe**	173	228	1.63	25.70	2.77	43.70
**South-East Asia**	348	505	0.06	12.30	0.11	20.91
**Western Pacific**	420	528	0.25	56.32	0.43	95.74
**Global total (females)**	**1709**	**1931**	**26**	**103**	**44**	**174**
**Males**						
	**Prevalent HSV-1 infection (any site)**		**Prevalent genital HSV-1 infection (assuming 50% of incident infections are genital from age 15)**		**Prevalent genital HSV-1 infection (assuming 85% of incident infections are genital from age 15)**	
**Region**	**Lower bound**	**Upper bound**	**Lower bound**	**Upper bound**	**Lower bound**	**Upper bound**
**Americas**	115	174.5	0.03	27.14	0.06	46.14
**Africa**	339^a^	359^a^	0.05^a^	0.14^a^	0.09^a^	0.23^a^
**Eastern Mediterranean**	164	225	0.04	15.13	0.06	25.71
**Europe**	153	213	0.36	27.84	0.60	47.33
**South-East Asia**	370	536	0.07	12.87	0.11	21.87
**Western Pacific**	448	565	0.27	59.03	0.45	100.34
**Global total (males)**	**1728**	**1965**	**33**	**113**	**56**	**192**

95% credible bounds for each regional set of estimates (by sex) were computed as follows. Firstly we sampled each pooled HSV-1 prevalence value 1000 times assuming a normal distribution, using the standard deviation derived from the meta-analysis. Next, we recalibrated λ and k for each set of sampled pooled prevalence values by region (by sex for the Americas and Europe). The resulting set of 1000 estimates was then sorted low-high for each estimate, sex and region of interest and the 2.5 and 97.5 percentile estimates extracted for the lower and upper uncertainty bounds. Region-specific estimates for genital HSV-1 are given to 2 d.p. due to some small numbers of infected individuals, but this level of accuracy is unlikely to be supported by the model. Note that stratified ranges do not sum to unstratified ranges.

^a^Credible range not reliable due to limited sampling of pooled prevalence.

## Discussion

An estimated 3709 million people globally aged 0–49 years were infected with prevalent HSV-1 in 2012. Assuming 50% of incident infections among 15-49-year-olds are genital, an estimated 140 million people were infected with prevalent genital HSV-1. The number of HSV-1 infections was highest for Africa, South-East Asia and Western Pacific, which had the largest population sizes. However, the majority of genital HSV-1 infections were in the Americas, Europe and Western Pacific, where HSV-1 infection continued to increase after adolescence. The prevalence of genital HSV-1 was highest for the Americas, which had the lowest HSV-1 prevalence on entering adolescence of all 6 regions.

Taken together with estimates of HSV-2 infection among adults globally, which were all considered to be genital, our new estimates of HSV-1-associated genital infection suggested that 544 million people had genital infection due to either viral type worldwide in 2012, assuming half of incident HSV-1 infections in adults are genital. This figure could be as high as 633 million people, if 85% of incident HSV-1 infections in adults are genital, as suggested in a recent prospective study(13). It is unclear how much of this infection is recognized, as only about 10–20% of HSV-2 infections are diagnosed[14,38,39] and the analogous proportion for HSV-1 is unknown. In adults and adolescents, genital herpes due to HSV-1 is associated with less frequent symptomatic disease recurrences than HSV-2[5,18,19]. Thus, disease burden will vary by region not only by the number of people with genital herpes, but also the relative proportions caused by HSV-2 versus HSV-1.

Although the burden of genital HSV-2 is high in Africa and moderate in South-East Asia, the available data suggest that genital HSV-1 is currently less likely to be an important public health problem in these regions. This is because HSV-1 acquisition here appeared to be high prior to adolescence and thus sexual debut, thereby protecting individuals in these regions from genital disease. However, the prevalence data on which the estimates were based were extremely sparse. For Africa, only one pooled prevalence estimate was available, with the remainder of the prevalence data for fitting coming from single studies. Genital HSV-1 could be much higher than estimated if HSV-1 infection continued after adolescence in these regions. Additionally, regional estimates could mask very different patterns of infection, including higher genital HSV-1, in individual countries or in specific settings.

Our estimates of HSV-1 are considerably hampered by small numbers of studies across all regions, and by issues of small sample size, quality of data and generalizability within studies. Estimates of the burden of genital HSV-1 are severely hampered by lack of data on the proportion of incident HSV-1 infection from adolescence that is genital. We attempted to account for this uncertainty by generating two sets of estimates for genital HSV-1: the first assuming a value of 50% for this proportion[36], and the second assuming a value of 85%[13]. These values are the best available, but based on very small samples and only US populations. In addition, the site of new HSV-1 infection can only be determined for symptomatic people, and a large proportion of infections are asymptomatic. The estimates assume that the proportions of new HSV-1 infections that are genital versus oral are the same for both symptomatic and asymptomatic people. If new genital HSV-1 infections are more likely than new oral HSV-1 infections to be symptomatic, the estimated proportion of all incident HSV-1 infections that are genital may be inflated. This proportion may also vary considerably by region, setting, age and sex, although this variability may be less important for regions like Africa and South-East Asia with apparently little HSV-1 acquisition after early adolescence. Although we computed uncertainty bounds around the estimates, these are unlikely to fully account for all variation in underlying prevalence, most notably because reliable bounds for the African estimates could not be calculated. Consideration of the estimate reliability in light of these important issues should accompany interpretation and application of these estimates.

Recent changes in the pattern of HSV-1 infection, i.e., decreasing rates of oral HSV-1 infection in childhood and increasing sexual transmission of HSV-1, mean that there may be cohort effects in prevalence data whereby older individuals have experienced higher historic rates of childhood infection and lower rates of sexual transmission. In the National Health and Nutrition Examination Surveys (NHANES) in the USA, the only nationally-representative general population surveys of HSV-1 prevalence repeated over a number of years, HSV-1 seroprevalence has been declining since the first surveys (1988–1994) and is continuing to decrease, with the largest decreases observed for adolescents[14,40]. We used only data from 2000 onwards (with the exception of Africa and South-East Asia), which minimized the influence of cohort effects to some extent. However this would not have removed the effect of all ongoing HSV-1 trends in the Americas and potentially other settings. The model in effect enables us to fit a function to smooth out observed prevalence, and then estimate the number of incident infections that would result from the fitted function, assuming that over the short period of one year prevalence does not change.

### Impact and recommendations

This is the first attempt to calculate the global burdens of all HSV-1 and genital HSV-1, and hence the first attempt to quantify the extent to which genital HSV-1 presents a public health problem across different regions. To date, prevention and control efforts against genital herpes have focussed almost exclusively on HSV-2. We show that this strategy is likely to be currently appropriate for Africa and South-East Asia, where genital HSV-1 infection is probably not a public health issue at this time. However, for other regions, most notably the Americas, Europe and Western Pacific, such a strategy would fail to address a substantial burden of genital HSV-1 infection. In addition to the consequences of genital HSV-1 infection, orolabial HSV-1 infection is important in its own right. Symptomatic recurrent orolabial herpes can range from a mere annoyance to severe disease in the setting of immunocompromise. In addition, HSV-1 is one of the most common causes of sporadic encephalitis, which is rare but devastating, with high morbidity and associated costs[6,7] and HSV-1 also contributes to neonatal infections and deaths.

Patterns of HSV-1 and HSV-2 infection, and their relative contribution to genital disease, are a product of multiple interactions. These include: different routes of transmission, different risk profiles by age, and the role of cross-immunity. Decreases in childhood HSV-1 infection and increases in orogenital sex have the potential to introduce genital HSV-1 as a public health issue in those regions where it is currently minimally present, since decreasing childhood-acquired immunity to HSV-1 (oral HSV-1 otherwise seems to protect against genital HSV-1) means more adults are at risk of acquiring genital HSV-1 through oral sex. Since HSV-1 infection does not seem to follow HSV-2[37], interventions that decrease HSV-2 infection could potentially lead to an increase in genital HSV-1, while a decrease in HSV-1 could lead to an increase in HSV-2 disease[36]. Other considerations for control programmes include: genital herpes due to HSV-1 is less likely to recur compared with HSV-2; the risk of neonatal herpes seems to be higher for HSV-1 than for HSV-2; and genital HSV-1 could in theory enhance HIV acquisition in a similar way to HSV-2 although the association is not understood.

Despite the limited availability of data informing these estimates, we hope to increase understanding of the global scope of HSV-1 infection, and guide development of future prevention efforts. In a recent Phase III trial, an HSV vaccine based on glycoprotein D2 failed to prevent HSV-2 infection and disease, but, encouragingly, did show significant efficacy against HSV-1-related infection and disease[41]. Efforts to develop new HSV vaccines are advancing[42]. There are currently a number of HSV vaccine candidates in the development pipeline, with several therapeutic (vaccines which work in those already infected to reduce viral shedding and disease) and prophylactic vaccines in Phase I and II trials, in conjunction with substantial advances in delivery systems, adjuvants and stimulation of mucosal immunity[42]. These estimates lay the groundwork for determining the potential impact of a vaccine against HSV and informing important vaccine characteristics. For the Americas, Europe and the Western Pacific, a vaccine would need to target both HSV types to prevent genital herpes. In Africa and South-East Asia, a vaccine targeting HSV-2 infection would address genital herpes but would need to be effective in the presence of HSV-1 infection if targeted to adolescents or would need to be an infant vaccine.

In summary, these WHO estimates show an enormous burden of HSV-1 globally, with regional variation in the age at which HSV-1 is acquired. Increased data on the epidemiology of HSV-1 are needed to strengthen the robustness of these estimates and provide a clearer picture in all regions as to how HSV-1 differs by age and sex. Furthermore, an improved understanding of the interaction between HSV-1 and HSV-2 at different anatomic sites on protective immunity is needed. Genital HSV-1 acquisition is lowest in regions such as Africa with the highest HIV rates, but understanding how HSV-1 affects HIV spread is critical given how common this infection is globally. It is hoped these estimates will be used to develop appropriate prevention messages, manage and counsel patients with symptomatic genital herpes, develop improved treatment regimens and diagnostic tests, and ultimately, develop HSV vaccines.

## Supporting Information

S1 Database Studies reporting HSV-1 seroprevalence and incidence in general populations identified from the current literature review. (XLSX)

S1 DOCUMENT Further details of the Methods. (DOCX)

S1 Reference List List of publications to accompany S1 Database. (DOCX)

S1 Table Region groupings. (DOCX)

S2 Table Region-specific HSV-1 prevalence criteria for use in the estimates. (Footnote to S2 Table) ^1^Age-stratified data used preferentially over sex-stratified data where not stratified by both simultaneously; ^2^Relaxing this restriction would not have had any effect on data availability.
(DOCX)

S3 Table Number of studies contributing HSV-1 prevalence to the 2012 estimates, by region. (Footnote to S3 Table) ^a^Not used in pooling since N = 1; ^b^Used in model fitting despite N = 1, due to poor data availability; ^c^Females; ^d^Males.
(DOCX)

S4 Table Pooled log odds of infection, τ^2^ values and I^2^ values, from the meta-analysis. (Footnote to S4 Table) N: number of observations; N≥2 required for pooling except for Africa; τ^2^: measure of between-study variance in log odds; I^2^: percentage of variation in study log odds due to between-study variation.
(DOCX)

S5 Table Test adjustor (sensitivity and specificity) values used, by assay type. (Footnote to S5 Table) ^a^Same value as Focus assumed; ^b^Source is manufacturer; ^c^FDA documentation. [1] Groen J, Van Dijk G, Niesters HG, Van Der Meijden WI, Osterhaus AD. Comparison of two enzyme-linked immunosorbent assays and one rapid immunoblot assay for detection of herpes simplex virus type 2-specific antibodies in serum. Journal of clinical microbiology. 1998;36(3):845–7. [2] Ribes JA, Smith A, Hayes M, Baker DJ, Winters JL. Comparative performance of herpes simplex virus type 1-specific serologic assays from MRL and Meridian Diagnostics. Journal of clinical microbiology. 2002;40(3):1071–2.
(DOCX)
