# Supplementary material for: Global and Regional Estimates of Prevalent and Incident Herpes Simplex Virus Type 1 Infections in 2012
Source: PLoS One. 2015 Oct 28;10(10):e0140765. doi: 10.1371/journal.pone.0140765 (PMC4624804; doi:10.1371/journal.pone.0140765)
Supplement: S1 DOCUMENT — (DOCX) [file pone.0140765.s002.docx]

Search strategy and selection criteria

PubMed and EMBASE databases were searched for potentially-relevant publications reporting HSV-1 prevalence and/or incidence and published from 2005 onwards. MeSH terms used in the PubMed search (date of search 12/02/2014) included “seroepidemiologic studies”, “prevalence”, “cross-sectional studies”, “incidence”, “cohort studies”, “follow-up studies”, “longitudinal studies”, “time factors”, “prospective studies” OR “survival analysis”; AND: “simplexvirus”, “herpes simplex”, “herpesvirus 1, human”, “herpesvirus 2, human”, “herpes genitalis”, “herpes labialis“ OR “stomatitis, herpetic”. Subject headings used in the EMBASE search (date of search 23/10/2013) were “Herpes simplex virus”, “Herpes simplex virus 1”, “Herpes simplex virus 2”, “herpes simplex”, “genital herpes” OR “herpes labialis”; AND: “seroepidemiology”, “incidence” OR “prevalence”; filters: human. No other restrictions were placed on the searches including with respect to language. Reference lists of key publications were also searched for additional publications with relevant data. Data from publications published prior to 2005 have been extracted previously[[1](#_ENREF_1),[2](#_ENREF_2)] and were also included if the selection criteria were met.

We defined HSV-1 prevalence as the percentage of individuals with type-specific IgG antibodies (in blood/serum) to HSV-1 cross-sectionally. HSV-1 incidence was defined as the rate or risk of IgG seroconversion to HSV-1. Where not given directly in tables or text, we calculated these from given numbers where possible, or took estimated values from graphs. Studies were required to give some detail of study location (at the minimum, the country or countries in which the study was conducted) and some information on age (mean, median or range). Prevalence and incidence rates were extracted both stratified and unstratifed by age and sex. If more than one publication presented findings from the same study, then unique data with the largest sample size were extracted (but all relevant publications listed). We used weighted or adjusted prevalence values where given. Where comparison results from more than one test were presented, we extracted those results from the assay or method considered to have the highest sensitivity and specificity. Equivocal samples, where not resolved by retesting, were excluded from both numerator and denominator.

Studies were excluded if participants were selected on the basis of having a medical condition. The rationale for this was that this condition may be associated with HSV infection, and/or prevalence in this group may not be generalizable to the general population. Excluded studies included studies of individuals with atopy, individuals undergoing transplantations, individuals with eye infections, and individuals with a genital or urinary tract infection. Additionally, studies were excluded if selection of study participants could lead to a bias in the subsequent estimates; for example, if participants were selected on the basis of having a history of cold sores. Where applicable, incidence rates were extracted only for those individuals in the control arm for interventions which might alter HSV-1 risk, and on an intention-to-treat basis.

Only prevalence values from general populations were retained for the analyses: data from specialised study populations were not used. Examples of such populations were men who have sex with men, STI clinic attendees, and commercial sex workers. We also did not use data from those studies where enrolment was based on a particular minority subpopulation.

Calculation of HSV-1 prevalence and incidence estimates

Estimates of the burden of prevalent and incident HSV-1 infection were generated for each of the 6 WHO regions: the Americas, Africa, Eastern Mediterranean, Europe, South-East Asia and Western Pacific (S1 Table). Age-stratified prevalence was used preferentially over unstratified prevalence. Specific regional criteria for use in the estimates were also applied depending on data availability by sex and study year for each region (S2 Table). In particular, only those prevalence values from studies from 2000 onwards were used, except for Africa and South-East Asia, where prevalence values from 1995 onwards were used due to poor data availability. Data availability allowed fitting by sex for the Americas and Europe only.

A sample size of 20 or more was required for use in the estimates; age strata with sample sizes smaller than this were combined and applied to both categories of age. Sample sizes for age strata where not given were estimated from the total sample size and widths of the age strata. The mean or median age was used where given. The mid-point was used for age reported in ranges. Age limits for age in ranges where not defined were assumed to extend for 10 years (e.g., <25 years → 15-24; ≥35 years → 35-44). This was applied in all cases regardless as to the study population (e.g., if the study included children or not); however the overall age range of the entire sample was used to delineate age strata where possible. Mean/median ages and mid-points of ages were then grouped as follows: 0-4; 5-9; 10-14; 15-19; 20-24; 25-29; 30-34; 35-39; 40-44 and 45-49 years.

We adjusted each prevalence value for the sensitivity and specificity of the assay used[[3](#_ENREF_3)], according to the package insert of the assay, USA Food and Drug Administration (FDA) documentation or published test performance[[4](#_ENREF_4),[5](#_ENREF_5)] and using the following equation, based on Bayes’ rule:

Corrected prevalence = (Reported prevalence + specificity - 1) / (sensitivity + specificity - 1)

with prevalence, sensitivity and specificity expressed as proportions. Where the assay had unknown sensitivity or specificity, sensitivity and specificity values for Focus were applied. Prevalence values of 100% or over generated as a consequence of adjustment were recoded as 99.9%.

For each region, pooled prevalence values by 5-year age group from 0-49 years (all regions), and by sex (the Americas and Europe only) were generated in Stata (Stata 13; StataCorp, College Station, Texas, USA). This was done using the metan command to pool the raw log odds of infection weighted by the standard error of the log odds of infection from individual included studies according to the age of each sample, using a random-effect model to allow for between- as well as within-study variation. Next we calibrated HSV-1 incidence and the maximum proportion able to be infected (to allow for saturation in prevalence below 100% where supported by data) by using maximum likelihood to fit a model with constant HSV-1 incidence by age to the pooled prevalence values[[6](#_ENREF_6)]. The model equation was as follows:

*F*(*a*) = *k* * (1 - e^(-λ *^ *^a^*^)^)

where *F*(*a*) is the proportion of individuals HSV-1 seropositive at age *a* in years (i.e., with prevalent HSV-1 infection), *k* is the maximum proportion of individuals able to be infected and λ is the force of infection per year. Although the force of infection is assumed to be constant across all ages, this is a per susceptible risk, and hence the number of new infections rapidly declines with age and prevalence is able to “level off”, while the parameter *k* allows prevalence to saturate below 100%. Specifically, fitting was done by using the Solver function in Excel to find those values of *k* and λ which maximized the value of:

$$ln\prod_{a} \left[ {F(a)}^{S(a) * P(a)}*{(1-F\left( a \right))}^{S(a) * (1-P\left( a \right))} \right]$$

where *a* is the mid-point of each 5-year age group, *S*(*a*) is the total sample size (from summing across all studies) and *P*(*a*) is the pooled HSV-1 prevalence.

Once λ and *k* were computed, the smoothed HSV-1 seroprevalence estimates by 5-year age group (and by sex where feasible) resulting from the model fits (*F*(*a*)) were then multiplied by the regional population size obtained from the United Nations Population Division for 2012[[7](#_ENREF_7)] to estimate the numbers of people with prevalent HSV-1 infection by region in 2012. The numbers of people with incident HSV-1 infection by region in 2012 were obtained by applying the model incidence to the population sizes remaining susceptible. Specifically, the numbers of new cases of HSV-1 infection at each single year of age, *I*(*a*), were calculated as:

*I*(*a)* = (*k* - *F*(*a*)) * λ * *N*(*a*)

where *N*(*a*) is the total number of individuals (i.e., regional population size) at age *a*. Estimates were then summed across ages for each 5-year age category. Model incidence was used rather than reported incidence due to a lack of reported incidence values across all ages and regions. Also, an advantage of the pooling and smoothing process is that calibrated incidence is less variable. Global estimates were obtained by summing estimates over all 6 regions.

The estimated prevalence of genital HSV-1 infection by single year of age *a* among individuals aged between 15 and 49 years was calculated from:

*F_g_(a)* = (*F(a)*-*F(15)*) * *p*_g_

where *F_g_(a)* is the proportion of individuals with prevalent genital HSV-1 infection at age *a* and *p*_g_ is the proportion of new HSV-1 infections in adulthood that are genital. The value of *p*_g_ is uncertain though some estimates are available. In a prospective study in the USA of HSV-2 seronegative individuals aged 17-79 years who were “high-risk” or in an HSV-2 serodiscordant relationship, 6 out of 12 symptomatic incident HSV-1 infections (out of a total of 19 incident HSV-1 infections) were associated with genital lesions (50%); the remaining symptomatic individuals had either orolabial lesions or pharyngitis[[8](#_ENREF_8)]. In HSV seronegative women aged 18-30 years who were in the control arm of the HERPEVAC Trial for Women in the USA, 28 out of 33 symptomatic incident HSV-1 infections (out of a total of 127 incident HSV-1 infections) were associated with either genital disease or both oral and genital disease (85%)[[9](#_ENREF_9)]. Note that since some women reported both oral and genital disease at the same time, some women were likely infected with both oral and genital HSV-1 infection concurrently. The proportion may vary between regions and different population groups. In the absence of further data to inform this parameter we did all analyses for genital HSV-1 infection firstly assuming that 50% of incident HSV-1 infections are genital from age 15 years, and then repeating the analyses but assuming this proportion is 85%.

Genital HSV-1 prevalence estimates were then applied to population sizes and summed across 5-year age bands. Estimates of the total number of genital herpes infections (HSV-1 or HSV-2) were calculated by summing estimates for HSV-1 and HSV-2[[10](#_ENREF_10)], and adjusting the estimates for co-infection by removing (genital HSV-1 prevalence * HSV-2 prevalence) proportionate infections to avoid double-counting. This assumed that previous HSV-2 infection is completely protective against subsequent genital HSV-1 infection, but that genital HSV-1 infection does not afford any protection against subsequent HSV-2 infection. If there were two routes to coinfection, rather than one, coinfection prevalence would be higher than that predicted by the product of the individual prevalences of HSV-2 and genital HSV-1 infection. (Conversely, the coinfection prevalence would be lower if genital HSV-1 did afford some protection against subsequent HSV-2, and therefore the total number of genital herpes infections would be even higher.) This was tested using a very simple deterministic compartmental model of genital HSV-1 and HSV-2.

Uncertainty analysis

We computed 95% credible bounds around the numbers of individuals with prevalent HSV-1 infection in 2012 (any site and genital only), as a function of uncertainty in the underlying prevalence data, as follows. First, we sampled each region-specific, age-specific pooled HSV-1 prevalence value assuming a normal distribution, and derived the standard deviation for each pooled prevalence value from the results of the meta-analysis. The exception was for Africa, where only one pooled prevalence value was calculated and sampled (with raw prevalence values from single studies additionally used for subsequent model fitting). Thus, sampling will incorporate only a fraction of the total variation in the underlying prevalence data, and as such, the uncertainty bounds for Africa will not be reliable. Next, we recalibrated λ and k for each set of sampled pooled prevalence values by region (by sex for the Americas and Europe) to obtain smoothed HSV-1 seroprevalence and estimates of the numbers infected as described above. This was done 1000 times. The resulting set of 1000 estimates was then sorted from low to high for each estimate, sex and region of interest and the 2.5 and 97.5 percentile estimates extracted for the lower and upper uncertainty bounds.

References

1. Looker KJ, Garnett GP, Schmid GP (2008) An estimate of the global prevalence and incidence of herpes simplex virus type 2 infection. Bull World Health Organ 86: 805-812, A.

2. Smith JS, Robinson NJ (2002) Age-specific prevalence of infection with herpes simplex virus types 2 and 1: a global review. J Infect Dis 186 Suppl 1: S3-28.

3. Kelsey JL, Thompson WD, Evans AS (1986) Methods in Observational Epidemiology (Monographs in Epidemiology & Biostatistics): Oxford University Press.

4. Groen J, Van Dijk G, Niesters HG, Van Der Meijden WI, Osterhaus AD (1998) Comparison of two enzyme-linked immunosorbent assays and one rapid immunoblot assay for detection of herpes simplex virus type 2-specific antibodies in serum. J Clin Microbiol 36: 845-847.

5. Ribes JA, Smith A, Hayes M, Baker DJ, Winters JL (2002) Comparative performance of herpes simplex virus type 1-specific serologic assays from MRL and Meridian Diagnostics. J Clin Microbiol 40: 1071-1072.

6. Muench H (1959) Catalytic models in epidemiology. Harvard University Press.

7. World Population Prospects: The 2012 Revision. 2013 ed. New York, 2013: United Nations, Department of Economic and Social Affairs, Population Division.

8. Langenberg AG, Corey L, Ashley RL, Leong WP, Straus SE (1999) A prospective study of new infections with herpes simplex virus type 1 and type 2. Chiron HSV Vaccine Study Group. N Engl J Med 341: 1432-1438.

9. Bernstein DI, Bellamy AR, Hook EWr, Levin MJ, Wald A, et al. (2013) Epidemiology, clinical presentation, and antibody response to primary infection with herpes simplex virus type 1 and type 2 in young women. Clin Infect Dis 56: 344-351.

10. Looker KJ, Magaret A, Turner KME, Vickerman P, Gottlieb SL, et al. (2015) Global estimates of prevalent and incident herpes simplex virus type 2 infections in 2012. PLoS One 1: e114989.
