# Supplementary material for: Global and Regional Estimates of Prevalent and Incident Herpes Simplex Virus Type 1 Infections in 2012
Source: PLoS One. 2015 Oct 28;10(10):e0140765. doi: 10.1371/journal.pone.0140765 (PMC4624804; doi:10.1371/journal.pone.0140765)
Supplement: S1 Reference List — (DOCX) [file pone.0140765.s003.docx]

1. Aiello, A.E., et al., The influence of latent viral infection on rate of cognitive decline over 4 years. J Am Geriatr Soc, 2006. 54(7): p. 1046-54.

2. Aiello, A.E., et al., Persistent infection, inflammation, and functional impairment in older Latinos. J Gerontol A Biol Sci Med Sci, 2008. 63(6): p. 610-8.

3. Aiello, A.E., H.O. Nguyen, and M.N. Haan, C-reactive protein mediates the effect of apolipoprotein E on cytomegalovirus infection. J Infect Dis, 2008. 197(1): p. 34-41.

4. Dowd, J.B., et al., Socioeconomic gradients in immune response to latent infection. Am J Epidemiol, 2008. 167(1): p. 112-20.

5. Arriaga-Demeza, R.C., et al., Different patterns of herpes simplex virus type 1 infection among college students from Cuernavaca, Mexico. Sex Health, 2008. 5(4): p. 365-7.

6. Benharrosh, J., et al., [Comparison of two ELISA tests to study the seroprevalence of herpes simplex 1 et 2 infection in a maternity near Paris]. Ann Biol Clin (Paris), 2008. 66(6): p. 665-70.

7. Brazzale, A.G., et al., Seroprevalence of herpes simplex virus type 1 and type 2 among the Indigenous population of Cape York, Far North Queensland, Australia. Sex Health, 2010. 7(4): p. 453-9.

8. Clemens, S.A. and C.K. Farhat, Seroprevalence of herpes simplex 1-2 antibodies in Brazil. Rev Saude Publica, 2010. 44(4): p. 726-34.

9. Cunningham, A.L., et al., Prevalence of infection with herpes simplex virus types 1 and 2 in Australia: a nationwide population based survey. Sex Transm Infect, 2006. 82(2): p. 164-8.

10. Page, A., et al., Upstairs and downstairs: socio-economic and gender interactions in herpes simplex virus type 2 seroprevalence in australia. Sex Transm Dis, 2009. 36(6): p. 344-9.

11. Davidovici, B.B., et al., Decline in the prevalence of antibodies to herpes simplex virus types 1 and 2 among Israeli young adults between 1984 and 2002. Sex Transm Dis, 2006. 33(11): p. 641-5.

12. Davidovici, B.B., et al., Seroprevalence of herpes simplex virus 1 and 2 and correlates of infection in Israel. J Infect, 2006. 52(5): p. 367-73.

13. Davidovici, B.B., et al., Comparison of the dynamics and correlates of transmission of Herpes Simplex Virus-1 (HSV-1) and Varicella-Zoster Virus (VZV) in a sample of the Israeli population. Eur J Epidemiol, 2007. 22(9): p. 641-6.

14. Doi, Y., et al., Seroprevalence of herpes simplex virus 1 and 2 in a population-based cohort in Japan. J Epidemiol, 2009. 19(2): p. 56-62.

15. Dolar, N., et al., Seroprevalence of herpes simplex virus type 1 and type 2 in Turkey. J Eur Acad Dermatol Venereol, 2006. 20(10): p. 1232-6.

16. Dordevic, H., [Serological response to herpes simplex virus type 1 and 2 infection among women of reproductive age]. Med Pregl, 2006. 59(11-12): p. 591-7.

17. Fife, K.H., et al., Incidence and prevalence of herpes simplex virus infections in adolescent women. Sex Transm Dis, 2006. 33(7): p. 441-4.

18. Haddow, L.J., et al., Herpes simplex virus type 2 (HSV-2) infection in women attending an antenatal clinic in the South Pacific island nation of Vanuatu. Sex Transm Dis, 2007. 34(5): p. 258-61.

19. Jafarzadeh, A., et al., The association between infection burden in Iranian patients with acute myocardial infarction and unstable angina. Acta Med Indones, 2011. 43(2): p. 105-11.

20. Janson, C., et al., The effect of infectious burden on the prevalence of atopy and respiratory allergies in Iceland, Estonia, and Sweden. J Allergy Clin Immunol, 2007. 120(3): p. 673-9.

21. Juhl, D., et al., Detection of herpes simplex virus DNA in plasma of patients with primary but not with recurrent infection: implications for transfusion medicine? Transfus Med, 2010. 20(1): p. 38-47.

22. Kaur, R., N. Gupta, and U.K. Baveja, Seroprevalence of HSV1 and HSV2 infections in family planning clinic attenders. J Commun Dis, 2005. 37(4): p. 307-9.

23. Kramer, M.A., et al., Ethnic differences in HSV1 and HSV2 seroprevalence in Amsterdam, the Netherlands. Euro Surveill, 2008. 13(24).

24. Kucera, P., et al., Seroepidemiology of herpes simplex virus type 1 and 2 in pregnant women in Switzerland: an obstetric clinic based study. Eur J Obstet Gynecol Reprod Biol, 2012. 160(1): p. 13-7.

25. Lin, H., et al., Herpes simplex virus infections among rural residents in eastern China. BMC Infect Dis, 2011. 11: p. 69.

26. Lupi, O., Prevalence and risk factors for herpes simplex infection among patients at high risk for HIV infection in Brazil. Int J Dermatol, 2011. 50(6): p. 709-13.

27. Mahjour, S.B., et al., Seroprevalence of human herpes simplex, hepatitis B and epstein-barr viruses in children with acute lymphoblastic leukemia in southern iran. Pathol Oncol Res, 2010. 16(4): p. 579-82.

28. Michos, A., et al., Association of allergic sensitization with infectious diseases burden in Roma and non-Roma children. Pediatr Allergy Immunol, 2011. 22(2): p. 243-8.

29. Mowry, E.M., et al., Vitamin D status and antibody levels to common viruses in pediatric-onset multiple sclerosis. Mult Scler, 2011. 17(6): p. 666-71.

30. Nabipour, I., et al., The association of metabolic syndrome and Chlamydia pneumoniae, Helicobacter pylori, cytomegalovirus, and herpes simplex virus type 1: the Persian Gulf Healthy Heart Study. Cardiovasc Diabetol, 2006. 5: p. 25.

31. Ozdemir, R., et al., [HSV-1 and HSV-2 seropositivity rates in pregnant women admitted to Izmir Ataturk Research and Training Hospital, Turkey]. Mikrobiyol Bul, 2009. 43(4): p. 709-11.

32. Page, W.F., et al., National estimates of seroincidence and seroprevalence for herpes simplex virus type 1 and type 2 among US military adults aged 18 to 29 years. Sex Transm Dis, 2012. 39(4): p. 241-50.

33. Papadogeorgakis, H., et al., Herpes simplex virus seroprevalence among children, adolescents and adults in Greece. Int J STD AIDS, 2008. 19(4): p. 272-8.

34. Patnaik, P., et al., Type-specific seroprevalence of herpes simplex virus type 2 and associated risk factors in middle-aged women from 6 countries: the IARC multicentric study. Sex Transm Dis, 2007. 34(12): p. 1019-24.

35. Patterson, J., et al., Genital HSV detection among HIV-1-infected pregnant women in labor. Infect Dis Obstet Gynecol, 2011. 2011: p. 157680.

36. Prasad, K.M., et al., Progressive gray matter loss and changes in cognitive functioning associated with exposure to herpes simplex virus 1 in schizophrenia: a longitudinal study. Am J Psychiatry, 2011. 168(8): p. 822-30.

37. Sauerbrei, A., et al., Seroprevalence of herpes simplex virus type 1 and type 2 in Thuringia, Germany, 1999 to 2006. Euro Surveill, 2011. 16(44).

38. Smith, J.S., et al., Type specific seroprevalence of HSV-1 and HSV-2 in four geographical regions of Poland. Sex Transm Infect, 2006. 82(2): p. 159-63.

39. Sun, Y., et al., An association of herpes simplex virus type 1 infection with type 2 diabetes. Diabetes Care, 2005. 28(2): p. 435-6.

40. Tedla, Y., et al., Serum antibodies to Toxoplasma gondii and Herpesvidae family viruses in individuals with schizophrenia and bipolar disorder: a case-control study. Ethiop Med J, 2011. 49(3): p. 211-20.

41. Vilibic-Cavlek, T., et al., Seroprevalence of TORCH infections in women of childbearing age in Croatia. J Matern Fetal Neonatal Med, 2011. 24(2): p. 280-3.

42. Vilibic-Cavlek, T., et al., Herpes simplex virus infection in the Croatian population. Scand J Infect Dis, 2011. 43(11-12): p. 918-22.

43. Xu, F., et al., Trends in herpes simplex virus type 1 and type 2 seroprevalence in the United States. JAMA, 2006. 296(8): p. 964-73.

44. Xu, F., et al., Seroprevalence of herpes simplex virus type 1 in children in the United States. J Pediatr, 2007. 151(4): p. 374-7.

45. Corona-Oregon, E., et al., Herpes simplex virus type 1: A possible agent of sexual transmission among university students. [Spanish]

Virus del herpes simplex tipo 1: Un posible agente de transmision sexual en poblacion universitaria. Gaceta Medica de Mexico, 2010. 146(2): p. 98-102.

46. Heiligenberg, M., et al., Seroprevalence and determinants of eight high-risk human papillomavirus types in homosexual men, heterosexual men, and women: A population-based study in Amsterdam. Sexually Transmitted Diseases, 2010. 37(11): p. 672-680.

47. Vahdat, K., et al., Concurrent increased high sensitivity C-reactive protein and chronic infections are associated with coronary artery disease: A population-based study. Indian Journal of Medical Sciences, 2007. 61(3): p. 135-143.

48. Vahdat, K., et al., Association of pathogen burden and hypertension: The persian gulf healthy heart study. American Journal of Hypertension, 2013. 26(9): p. 1140-1147.

49. Kumar, N., et al., Human herpesvirus 8 genoprevalence in populations at disparate risks of Kaposi's sarcoma. Journal of Medical Virology, 2007. 79(1): p. 52-59.

50. Tayyebi, D. and S. Sharifi, Seroepidemiology of infection with herpes simplex virus types 1 and 2 (HSV1 and HSV2) among asymptomatic university students attending Islamic Azad university of Kazeroun, Southwest of Iran. Iranian Journal of Clinical Infectious Diseases, 2010. 5(2): p. 84-88.

51. Wang, H., et al., Antibodies to infectious agents and the positive symptom dimension of subclinical psychosis: The TRAILS study. Schizophr Res, 2011. 129(1): p. 47-51.

52. Bernstein, D.I., et al., Epidemiology, clinical presentation, and antibody response to primary infection with herpes simplex virus type 1 and type 2 in young women. Clin Infect Dis, 2013. 56(3): p. 344-51.

53. Howard, M., et al., Regional distribution of antibodies to herpes simplex virus type 1 (HSV-1) and HSV-2 in men and women in Ontario, Canada. J Clin Microbiol, 2003. 41(1): p.84-9.

54. Stanberry, L.R., et al., Longitudinal risk of herpes simplex virus (HSV) type 1, HSV type 2, and cytomegalovirus infections among young adolescent girls. Clin Infect Dis, 2004. 39(10): p.1433-1438.

55. Dan, M., et al., Prevalence and risk factors for herpes simplex virus type 2 infection among pregnant women in Israel. Sex Transm Dis, 2003. 30(11): p.835-8.

56. Smith, J.S., et al., Herpes simplex virus-2 as a human papillomavirus cofactor in the etiology of invasive cervical cancer. J Natl Cancer Inst, 2002. 94(21): p.1604-13.

57. Cowan, F.M., et al., Seroepidemiological study of herpes simplex virus types 1 and 2 in Brazil, Estonia, India, Morocco, and Sri Lanka. Sex Transm Infect, 2003. 79(4): p.286-90.

58. Mihret, W., et al., Herpes simplex virus type 2 seropositivity among urban adults in Africa: results from two cross-sectional surveys in Addis Ababa, Ethiopia. Sex Transm Dis, 2002. 29(3): p.175-81.

59. Gwanzura, L., et al., The prevalence of Herpes simplex virus type-2 infection in blood donors in Harare, Zimbabwe. Cent Afr J Med, 2002. 48(3-4): p.38-42.

60. Alanen, A., et al., Seroprevalence, incidence of prenatal infections and reliability of maternal history of varicella zoster virus, cytomegalovirus, herpes simplex virus and parvovirus B19 infection in South-Western Finland. Bjog, 2005. 112(1): p.50-6.

61. Le Donne, M., et al., Sero-prevalence of cytomegalovirus, rubella, herpes simplex virus, varicella zoster virus, measles, parvovirus B19 and adenovirus in women with spontaneous abortion. Italian Journal of Gynaecology & Obstetrics, 2005. 17(1): p.29-35.

62. Madhavan, H.N. and K. Priya, The diagnostic significance of enzyme linked immuno-sorbent assay for herpes simplex, varicella zoster and cytomegalovirus retinitis. Indian J Ophthalmol, 2003. 51(1): p.71-5.

63. Mbopi-Keou, F.X., et al., Interactions between herpes simplex virus type 2 and human immunodeficiency virus type 1 infection in African women: opportunities for intervention. J Infect Dis, 2000. 182: p.1090-6.

64. Ghebrekidan, H. et al., Prevalence of herpes simplex virus types 1 and 2, cytomegalovirus, and varicella-zoster virus infections in Eritrea. J Clin Virol, 1999. 12: p.53-64.

65. Ruiz J.D., et al., Prevalence of HIV infection, sexually transmitted diseases, and hepatitis and related risk behavior in young women living in low-income neighborhoods of northern California. West J Med, 2000. 172: p.368-73.

66. Bogaerts J., et al., Sexually transmitted infections among married women in Dhaka, Bangladesh: unexpected high prevalence of herpes simplex type 2 infection. Sex Transm Infect 2001. 77: p.114-9.

67. Waubant, E., et al., Common viruses associated with lower pediatric multiple sclerosis risk. Neurology, 2011. 76(23): p. 1989-95.

68. Steptoe, A., et al., Socioeconomic status, pathogen burden and cardiovascular disease risk. Heart, 2007. 93(12): p. 1567-70.

69. Zhu, J., et al., Prevalence and persistence of antibodies to herpes viruses, Chlamydia pneumoniae and Helicobacter pylori in Alaskan Eskimos: the GOCADAN Study. Clin Microbiol Infect, 2006. 12(2): p. 118-22.
