# Supplementary material for: Global and Regional Estimates of Prevalent and Incident Herpes Simplex Virus Type 1 Infections in 2012
Source: PLoS One. 2015 Oct 28;10(10):e0140765. doi: 10.1371/journal.pone.0140765 (PMC4624804; doi:10.1371/journal.pone.0140765)
Supplement: S1 Table — (DOCX) [file pone.0140765.s004.docx]

| **Africa** |
| --- |
| Algeria, Angola, Benin, Botswana, Burkina Faso, Burundi, Cameroon, Cape Verde, Central African Republic, Chad, Comoros, Congo, Côte d'Ivoire, Democratic Republic of the Congo, Equatorial Guinea, Eritrea, Ethiopia, Gabon, Gambia, Ghana, Guinea, Guinea-Bissau, Kenya, Lesotho, Liberia, Madagascar, Malawi, Mali, Mauritania, Mauritius, Mozambique, Namibia, Niger, Nigeria, Rwanda, Sao Tome and Principe, Senegal, Seychelles, Sierra Leone, South Africa, South Sudan, Swaziland, Togo, Uganda, United Republic of Tanzania, Zambia, Zimbabwe |
| **Americas** |
| Antigua and Barbuda, Argentina, Bahamas, Barbados, Belize, Bolivia, Brazil, Canada, Chile, Colombia, Costa Rica, Cuba, Dominica, Dominican Republic, Ecuador, El Salvador, Grenada, Guatemala, Guyana, Haiti, Honduras, Jamaica, Mexico, Nicaragua, Panama, Paraguay, Peru, Saint Kitts and Nevis, Saint Lucia, Saint Vincent and the Grenadines, Suriname, Trinidad and Tobago, United States of America, Uruguay, Venezuela (Bolivarian Republic of) |
| **Eastern Mediterranean** |
| Afghanistan, Bahrain, Djibouti, Egypt, Iran (Islamic Republic of), Iraq, Jordan, Kuwait, Lebanon, Libyan Arab Jamahiriya, Morocco, Oman, Pakistan, Qatar, Saudi Arabia, Somalia, Sudan, Syrian Arab Republic, Tunisia, United Arab Emirates, Yemen |
| **Europe** |
| Albania, Andorra, Armenia, Austria, Azerbaijan, Belarus, Belgium, Bosnia and Herzegovina, Bulgaria, Croatia, Cyprus, Czech Republic, Denmark, Estonia, Finland, France, Georgia, Germany, Greece, Hungary, Iceland, Ireland, Israel, Italy, Kazakhstan, Kyrgyzstan, Latvia, Lithuania, Luxembourg, Malta, Monaco, Montenegro, Netherlands, Norway, Poland, Portugal, Republic of Moldova, Romania, Russian Federation, San Marino, Serbia, Slovakia, Slovenia, Spain, Sweden, Switzerland, Tajikistan, The former Yugoslav Republic of Macedonia, Turkey, Turkmenistan, Ukraine, United Kingdom of Great Britain and Northern Ireland, Uzbekistan |
| **South-East Asia** |
| Bangladesh, Bhutan, Democratic People's Republic of Korea, India, Indonesia, Maldives, Myanmar, Nepal, Sri Lanka, Thailand, Timor-Leste |
| **Western Pacific** |
| Australia, Brunei Darussalam, Cambodia, China, Cook Islands, Fiji, Japan, Kiribati, Lao People's Democratic Republic, Malaysia, Marshall Islands, Micronesia (Federated States of), Mongolia, Nauru, New Zealand, Niue, Palau, Papua New Guinea, Philippines, Republic of Korea, Samoa, Singapore, Solomon Islands, Tonga, Tuvalu, Vanuatu, Viet Nam |
