# Supplementary material for: Global and Regional Estimates of Prevalent and Incident Herpes Simplex Virus Type 1 Infections in 2012
Source: PLoS One. 2015 Oct 28;10(10):e0140765. doi: 10.1371/journal.pone.0140765 (PMC4624804; doi:10.1371/journal.pone.0140765)
Supplement: S2 Table — (Footnote to S2 Table) 1Age-stratified data used preferentially over sex-stratified data where not stratified by both simultaneously; 2Relaxing this restriction would not have had any effect on data availability. (DOCX) [file pone.0140765.s005.docx]

| **Region** | **Study years included** | **Age-stratified estimates produced?** | **Sex-stratified estimates produced?** | **Data inclusion limited to data with known sample size and finite age limits only?** | **Notes** |
| --- | --- | --- | --- | --- | --- |
| **Americas** | 2000-present | Yes  (age-stratified data used preferentially over unstratified data) | Yes;  unstratified data not used | Yes | -- |
| **Africa** | 1995-present | Yes  (age-stratified data used preferentially over unstratified data) | No  (sex-stratified data used preferentially over unstratified data^1^) | Yes^2^ | Individual (N=1) raw prevalence values additionally used for fitting due to poor data availability |
| **Eastern Mediterranean** | 2000-present | Yes  (age-stratified data used preferentially over unstratified data) | No  (sex-stratified data used preferentially over unstratified data^1^) | No | -- |
| **Europe** | 2000-present | Yes  (age-stratified data used preferentially over unstratified data) | Yes;  unstratified data not used | Yes | -- |
| **South-East Asia** | 1995-present | Yes  (age-stratified data used preferentially over unstratified data) | No  (sex-stratified data used preferentially over unstratified data^1^) | No | -- |
| **Western Pacific** | 2000-present | Yes  (age-stratified data used preferentially over unstratified data) | No  (sex-stratified data used preferentially over unstratified data^1^) | Yes | -- |

^1^Age-stratified data used preferentially over sex-stratified data where not stratified by both simultaneously; ^2^Relaxing this restriction would not have had any effect on data availability.
