# Supplementary material for: Global and Regional Estimates of Prevalent and Incident Herpes Simplex Virus Type 1 Infections in 2012
Source: PLoS One. 2015 Oct 28;10(10):e0140765. doi: 10.1371/journal.pone.0140765 (PMC4624804; doi:10.1371/journal.pone.0140765)
Supplement: S3 Table — (Footnote to S3 Table) aNot used in pooling since N = 1; bUsed in model fitting despite N = 1, due to poor data availability; cFemales; dMales. (DOCX) [file pone.0140765.s006.docx]

| **Region** | **Sex** | **Number of studies contributing to estimate** | | | | | | | | | | **% of world population in region by sex** | **Countries included** |
| --- | --- | --- | --- | --- | --- | --- | --- | --- | --- | --- | --- | --- | --- |
|  |  | **0-4 years** | **5-9 years** | **10-14 years** | **15-19 years** | **20-24 years** | **25-29 years** | **30-34 years** | **35-39 years** | **40-44 years** | **45-49 years** |  |  |
| **Americas** | F | 0 | 0 | 1^a^ | 7 | 9 | 3 | 2 | 3 | 2 | 1^a^ | 13.3 | Canada, Mexico and United States of America |
|  | M | 0 | 0 | 0 | 4 | 5 | 2 | 1^a^ | 2 | 1^a^ | 1^a^ | 12.8 | Canada, Mexico and United States of America |
| **Africa** | Both | 1^b^ | 1^b^ | 0 | 0 | 1^b^ | 4 | 1^b^ | 1^b^ | 0 | 0 | 14.9^c^;  14.4^d^ | Eritrea, Ethiopia, Central African Republic and Zimbabwe |
| **Eastern Mediterranean** | Both | 1^a^ | 2 | 1^a^ | 1^a^ | 2 | 1^a^ | 1^a^ | 1^a^ | 2 | 2 | 9.3^c^;  9.5^d^ | Iran (Islamic Republic of) and Morocco |
| **Europe** | F | 1^a^ | 2 | 2 | 1^a^ | 5 | 9 | 9 | 1^a^ | 2 | 1^a^ | 11.0 | Croatia, Finland, France, Germany, Greece, Israel, Italy, Poland, Serbia, Switzerland and Turkey |
|  | M | 1^a^ | 2 | 2 | 1^a^ | 3 | 4 | 1^a^ | 2 | 1^a^ | 1^a^ | 10.7 | Germany, Greece, and Poland |
| **South-East Asia** | Both | 2 | 3 | 2 | 1^a^ | 3 | 1^a^ | 4 | 1^a^ | 2 | 0 | 27.2^c^;  27.6^d^ | Bangladesh, India, Sri Lanka and Thailand |
| **Western Pacific** | Both | 0 | 2 | 2 | 2 | 5 | 2 | 3 | 4 | 3 | 4 | 24.3^c^; 25.0^d^ | Australia, China and Japan |

^a^Not used in pooling since N=1; ^b^Used in model fitting despite N=1, due to poor data availability; ^c^Females; ^d^Males.
