# Supplementary material for: Global and Regional Estimates of Prevalent and Incident Herpes Simplex Virus Type 1 Infections in 2012
Source: PLoS One. 2015 Oct 28;10(10):e0140765. doi: 10.1371/journal.pone.0140765 (PMC4624804; doi:10.1371/journal.pone.0140765)
Supplement: S4 Table — (Footnote to S4 Table) N: number of observations; N≥2 required for pooling except for Africa; τ2: measure of between-study variance in log odds; I2: percentage of variation in study log odds due to between-study variation. (DOCX) [file pone.0140765.s007.docx]

| **Americas** | | | | | | | | | | | |
| --- | --- | --- | --- | --- | --- | --- | --- | --- | --- | --- | --- |
| **Females** | | | | | | **Males** | | | | | |
| **Age group (years)** | **N** | **Total sample size** | **Pooled log odds of infection** | **τ^2^** | **I^2^** | **Age group (years)** | **N** | **Total sample size** | **Pooled log odds of infection** | **τ^2^** | **I^2^** |
| **0-4** | 0 | 0 |  |  |  | **0-4** | 0 | 0 |  |  |  |
| **5-9** | 0 | 0 |  |  |  | **5-9** | 0 | 0 |  |  |  |
| **10-14** | 1 | 174 |  |  |  | **10-14** | 0 | 0 |  |  |  |
| **15-19** | 7 | 2929 | -0.216 | 0.1034 | 82.2% | **15-19** | 4 | 2701 | -0.696 | 0.0622 | 72.9% |
| **20-24** | 9 | 2693 | 0.368 | 0.3319 | 93.9% | **20-24** | 5 | 1284 | -0.377 | 0.1303 | 85.2% |
| **25-29** | 3 | 1470 | 0.276 | 0.0822 | 65.0% | **25-29** | 2 | 1133 | 0.010 | 0.0154 | 38.5% |
| **30-34** | 2 | 105 | 0.995 | 0.0000 | 0.0% | **30-34** | 1 | 92 |  |  |  |
| **35-39** | 3 | 1341 | 0.729 | 0.0191 | 25.3% | **35-39** | 2 | 1090 | 0.475 | 0.0536 | 67.3% |
| **40-44** | 2 | 105 | 1.456 | 0.7327 | 83.2% | **40-44** | 1 | 75 |  |  |  |
| **45-49** | 1 | 1101 |  |  |  | **45-49** | 1 | 1094 |  |  |  |
| **Africa** | | | | | | **Eastern Mediterranean** | | | | | |
| **Age group (years)** | **N** | **Total sample size** | **Pooled log odds of infection** | **τ^2^** | **I^2^** | **Age group (years)** | **N** | **Total sample size** | **Pooled log odds of infection** | **τ^2^** | **I^2^** |
| **0-4** | 1 | 54 | 0.294 | -- | -- | **0-4** | 1 | 215 | -- | -- | -- |
| **5-9** | 1 | 84 | 3.690 | -- | -- | **5-9** | 2 | 359 | 0.798 | 0.693 | 95.3% |
| **10-14** | 0 | 0 | -- | -- | -- | **10-14** | 1 | 269 | -- | -- | -- |
| **15-19** | 0 | 0 | -- | -- | -- | **15-19** | 1 | 269 | -- | -- | -- |
| **20-24** | 1 | 299 | 3.042 | -- | -- | **20-24** | 2 | 360 | 1.417 | 0.1031 | 73.9% |
| **25-29** | 4 | 919 | 3.399 | 0.3940 | 68.9% | **25-29** | 1 | 539 | -- | -- | -- |
| **30-34** | 1 | 80 | 6.907 | -- | -- | **30-34** | 1 | 269 | -- | -- | -- |
| **35-39** | 1 | 45 | 1.643 | -- | -- | **35-39** | 1 | 269 | -- | -- | -- |
| **40-44** | 0 | 0 | -- | -- | -- | **40-44** | 2 | 438 | 5.013 | 1.0048 | 32.3% |
| **45-49** | 0 | 0 | 0.294 | -- | -- | **45-49** | 2 | 1791 | 1.847 | 0.0057 | 37.3% |
| **Europe** | | | | | | | | | | | |
| **Females** | | | | | | **Males** | | | | | |
| **Age group (years)** | **N** | **Total sample size** | **Pooled log odds of infection** | **τ^2^** | **I^2^** | **Age group (years)** | **N** | **Total sample size** | **Pooled log odds of infection** | **τ^2^** | **I^2^** |
| **0-4** | 1 | 60 | -- | -- | -- | **0-4** | 1 | 90 | -- | -- | -- |
| **5-9** | 2 | 138 | -0.467 | 0.0284 | 31.30% | **5-9** | 2 | 162 | -0.432 | 0.2682 | 83.20% |
| **10-14** | 2 | 152 | 0.106 | 0.0000 | 0.00% | **10-14** | 2 | 148 | 0.254 | 0.0000 | 0.00% |
| **15-19** | 1 | 89 | -- | -- | -- | **15-19** | 1 | 61 | -- | -- | -- |
| **20-24** | 5 | 624 | 1.344 | 0.3754 | 86.1% | **20-24** | 3 | 195 | 0.414 | 0.4021 | 84.4% |
| **25-29** | 9 | 1652 | 1.844 | 0.7396 | 92.2% | **25-29** | 4 | 737 | 0.763 | 0.0511 | 58.9% |
| **30-34** | 9 | 4470 | 1.298 | 0.7249 | 98.1% | **30-34** | 1 | 72 | -- | -- | -- |
| **35-39** | 1 | 36 | -- | -- | -- | **35-39** | 2 | 1103 | 1.446 | 0.6045 | 93.5% |
| **40-44** | 2 | 155 | 1.349 | 0.0000 | 0.0% | **40-44** | 1 | 51 | -- | -- | -- |
| **45-49** | 1 | 22 | -- | -- | -- | **45-49** | 1 | 30 | -- | -- | -- |
| **South-East Asia** | | | | | | **Western Pacific** | | | | | |
| **Age group (years)** | **N** | **Total sample size** | **Pooled log odds of infection** | **τ^2^** | **I^2^** | **Age group (years)** | **N** | **Total sample size** | **Pooled log odds of infection** | **τ^2^** | **I^2^** |
| **0-4** | 2 | 544 | -0.508 | 0.0000 | 0.0% | **0-4** | 0 | 0 | -- | -- | -- |
| **5-9** | 3 | 758 | 0.218 | 0.1896 | 90.8% | **5-9** | 2 | 115 | 0.406 | 0.0000 | 0.0% |
| **10-14** | 2 | 679 | 1.049 | 0.0000 | 0.0% | **10-14** | 2 | 109 | 1.241 | 0.0000 | 0.0% |
| **15-19** | 1 | 293 | -- | -- | -- | **15-19** | 2 | 167 | 1.510 | 0.2320 | 72.2% |
| **20-24** | 3 | 112 | -0.661 | 0.0412 | 24.8% | **20-24** | 5 | 385 | 0.791 | 1.2383 | 92.9% |
| **25-29** | 1 | 586 | -- | -- | -- | **25-29** | 2 | 247 | 2.242 | 0.2506 | 71.4% |
| **30-34** | 4 | 401 | 0.021 | 1.7475 | 95.2% | **30-34** | 3 | 353 | 2.708 | 0.2698 | 61.1% |
| **35-39** | 1 | 293 | -- | -- | -- | **35-39** | 4 | 611 | 1.337 | 2.0217 | 97.4% |
| **40-44** | 2 | 368 | 0.960 | 1.4824 | 97.3% | **40-44** | 3 | 309 | 4.193 | 0.0000 | 0.0% |
| **45-49** | 0 | 0 | -- | -- | -- | **45-49** | 4 | 582 | 1.707 | 1.0897 | 95.0% |

N: number of observations; N≥2 required for pooling except for Africa; τ^2^: measure of between-study variance in log odds; I^2^: percentage of variation in study log odds due to between-study variation.
