# Supplementary material for: Global and Regional Estimates of Prevalent and Incident Herpes Simplex Virus Type 1 Infections in 2012
Source: PLoS One. 2015 Oct 28;10(10):e0140765. doi: 10.1371/journal.pone.0140765 (PMC4624804; doi:10.1371/journal.pone.0140765)
Supplement: S5 Table — (Footnote to S5 Table) aSame value as Focus assumed; bSource is manufacturer; cFDA documentation. [1] Groen J, Van Dijk G, Niesters HG, Van Der Meijden WI, Osterhaus AD. Comparison of two enzyme-linked immunosorbent assays and one rapid immunoblot assay for detection of herpes simplex virus type 2-specific antibodies in serum. Journal of clinical microbiology. 1998;36(3):845–7. [2] Ribes JA, Smith A, Hayes M, Baker DJ, Winters JL. Comparative performance of herpes simplex virus type 1-specific serologic assays from MRL and Meridian Diagnostics. Journal of clinical microbiology. 2002;40(3):1071–2. (DOCX) [file pone.0140765.s008.docx]

| **Manufacturer** | **Assay name** | **Number of prevalence values using assay** | **Sensitivity (%)** | **Specificity (%)** |
| --- | --- | --- | --- | --- |
| **Biokit** | Bioelisa HSV-1 IgG | 4 | Unknown^a^ | Unknown^a^ |
| **Chiron** | Rapid Immunoblot Assay (RIBA) | 1 | 99.2[[1](#_ENREF_1)] | 97.1[[1](#_ENREF_1)] |
| **Diagnostic Bioprobes** | HSV 1&2 IgG | 3 | 98%^b^ | 98%^b^ |
| **Euroimmun** | Anti-HSV-1 ELISA (IgG) Kit | 1 | 98.1%^c^ | 77.8%^c^ |
| **Focus Diagnostics** | HerpeSelect 1 and 2 Immunoblot IgG | 58 | 99.3%^b^ | 95.1%^b^ |
| **Focus Diagnostics/Euroimmun** | HerpeSelect 1 and 2 Immunoblot IgG/ Anti-HSV-1 ELISA (IgG) Kit | 22 | 99.3%^b^ | 95.1%^b^ |
| **Generic** | -- | 8 | Unknown^a^ | Unknown^a^ |
| **Meridian Diagnostics (formerly Gull)** | Type-Specific HSV-1 IgG ELISA | 3 | 98.8%[[2](#_ENREF_2)] | 99.0%[[2](#_ENREF_2)] |
| **Euro Diagnostica** | Herpesscan | 12 | Unknown^a^ | Unknown^a^ |
| **--** | Immunoblot | 4 | Unknown^a^ | Unknown^a^ |
| **GenBio** | ImmunoDOT | 8 | 95%^b^ | Unknown^a^ |
| **MRL Diagnostics** | Immunoblot IgG Assay | 1 | 98.2%[[2](#_ENREF_2)] | 93.8%[[2](#_ENREF_2)] |
| **MRL/Meridian Diagnostics (formerly Gull)** | Immunoblot IgG Assay/ Type-Specific HSV-1 IgG ELISA | 27 | 98.8%[[2](#_ENREF_2)] | 99.0%[[2](#_ENREF_2)] |
| **DRG Diagnotics (formerly Novum Diagnostica)** | HSV-1&-2 IgG ELISA | 4 | Unknown^a^ | Unknown^a^ |
| **Radim** | Herpes S.V. type 1 IgG | 5 | 97.6%^b^ | 100%^b^ |
| **Savyon** | SeroHSV1 | 1 | Unknown^a^ | Unknown^a^ |
| **Quest Diagnostics (formerly UW)** | HSV Western Blot | 2 | 100% | 100% |

^a^Same value as Focus assumed; ^b^Source is manufacturer; ^c^FDA documentation.

1. Groen J, Van Dijk G, Niesters HG, Van Der Meijden WI, Osterhaus AD (1998) Comparison of two enzyme-linked immunosorbent assays and one rapid immunoblot assay for detection of herpes simplex virus type 2-specific antibodies in serum. J Clin Microbiol 36: 845-847.

2. Ribes JA, Smith A, Hayes M, Baker DJ, Winters JL (2002) Comparative performance of herpes simplex virus type 1-specific serologic assays from MRL and Meridian Diagnostics. J Clin Microbiol 40: 1071-1072.
